# Supplementary material for: MetaRibo-Seq measures translation in microbiomes
Source: Nat Commun. 2020 Jun 29;11:3268. doi: 10.1038/s41467-020-17081-z (PMC7324362; doi:10.1038/s41467-020-17081-z)
Supplement: Supplementary file 10 — Supplementary Data 7 [file 41467_2020_17081_MOESM10_ESM.zip › File2/Confidence_High_Taxonomy/137605_out.krona.html]

Javascript must be enabled to view this page.

members
magnitude
magnitudeUnassigned
count
unassigned
taxon
rank

137605\_out

19

19
superkingdom
2

1239
1
phylum

SRS077194\_contig\_number\_5378
19

17
class
186801

17
order
186802

family
186804
2

species
1520829

SRS011302\_contig\_number\_contig-100\_3399.3399SRS021484\_contig\_number\_1362
2

15

SRS017007\_contig\_number\_contig-100\_12963.12963SRS020628\_contig\_number\_contig-100\_4537.4537SRS064014\_contig\_number\_contig-100\_3465.3465SRS144014\_contig\_number\_contig-100\_11984.11984
4
family
543314

3
genus
2060094

2507160
species

SRS023958\_contig\_number\_268SRS104704\_contig\_number\_1203SRS143310\_contig\_number\_11520
3

1
86331
genus


SRS049389\_contig\_number\_194
1
86332
species


SRS019696\_contig\_number\_826SRS057692\_contig\_number\_1103SRS058693\_contig\_number\_2935SRS104087\_contig\_number\_14556SRS143529\_contig\_number\_24624
5
species
35517

species
143393
2

SRS017209\_contig\_number\_4174SRS049253\_contig\_number\_3940


SRS017191\_contig\_number\_119
1
2292891
species
